# Infections and their prognostic significance before diagnosis of chronic lymphocytic leukemia, non-Hodgkin lymphoma, or multiple myeloma

**DOI:** 10.1038/s41416-024-02816-2

**Published:** 2024-08-22

**Authors:** Esben Packness, Olafur Birgir Davidsson, Klaus Rostgaard, Michael Asger Andersen, Emelie Curovic Rotbain, Carsten Utoft Niemann, Christian Brieghel, Henrik Hjalgrim

**Affiliations:** 1 Department of Hematology, Rigshospitalet, University Hospital of Copenhagen, Copenhagen, Denmark; 2 Hematology, Danish Cancer Institute, Copenhagen, Denmark; 3 Department of Epidemiology Research, Statens Serum Institut, Copenhagen, Denmark; 4 Department of Clinical Pharmacology, Bispebjerg and Frederiksberg Hospital, Copenhagen, Denmark; 5 Department of Hematology, Roskilde Hospital, University Hospital of Zealand, Copenhagen, Denmark; 6 Department of Clinical Medicine, University of Copenhagen, Copenhagen, Denmark

**Keywords:** B-cell lymphoma, Risk factors

## Abstract

**Background:**

Immunodeficiency is a shared feature of B cell malignancies. The risk of infections and their prognostic significance after diagnosis are well characterized, but, conversely, less is known about prediagnostic infections in these domains.

**Methods:**

In matched case-control analyzes, using Danish nationwide registers, we assessed the rate of prediagnostic infections in chronic lymphocytic leukemia (CLL), diffuse large B cell lymphoma (DLBCL), multiple myeloma (MM), follicular lymphoma (FL), marginal zone lymphoma (MZL), and lymphoplasmacytic lymphoma (LPL). Survival analyzes of data from clinical registers were then used to determine the effect of infections in the year preceding diagnosis on overall survival. To yield results for as many patients as possible, antimicrobial prescriptions were used as surrogates for infections.

**Results:**

The nationwide and clinical registers comprised 30,389 patients, accumulating 213,649 antimicrobial prescriptions, and 18,560 patients accumulating 107,268 prescriptions, respectively. The relative risk of infections was increased up to 15 years prior to diagnosis of malignancy and markedly increased in the year just prior to diagnosis. More than two antimicrobials within one year prior to diagnosis were associated with significantly shorter overall survival, independently of known prognostic factors.

**Conclusion:**

Patients with B cell-derived malignancies exhibit marked immunodeficiency several years prior to diagnosis such that different disease subtypes demonstrate both overlapping and distinct trends in infection risk preceding diagnosis. Moreover, multiple infections within the year preceding diagnosis are independently associated with shorter overall survival for all the examined malignancies.

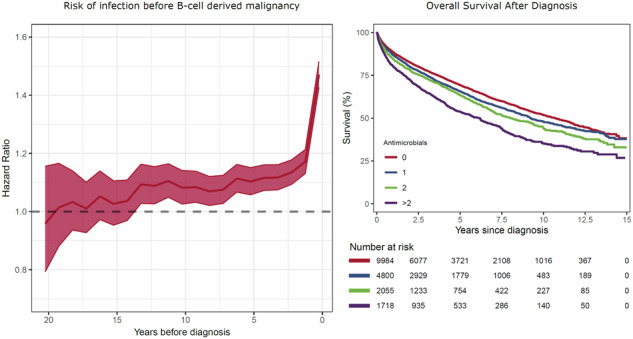

## Introduction

B cell-derived malignancies are typically accompanied by immune dysfunction and are associated with an increased risk of infections [1–4]. Upon diagnosis, the already impaired immune function is subject to further decline, which can be ascribed to the progression of lymphoma-associated immunodeficiency that is further aggravated by treatment once initiated. Therefore, it comes as no surprise that infections are among the main determinants of outcomes in patients with B cell-derived malignancies, generally conferring highly increased mortality in this patient population [2, 5–7].

In a recent study of chronic lymphocytic leukemia (CLL), we observed that individuals diagnosed with CLL demonstrated an increased use of antimicrobials, and thus risk of infections, relative to healthy controls starting more than 5 years before diagnosis [8]. Furthermore, approaching diagnosis, the risk of infections increased markedly, likely reflecting a sudden worsening of immune function. This suggested two immunological phenomena at play in these patients. First, a prolonged period of modestly increased risk of infections, and subsequently, the ostensible emergence of a malignancy resulting in more profound immune disruption.

In the present investigation, we continued this line of inquiry, extending our analyzes to include other B cell-derived malignancies and exploring the clinical significance of prediagnostic infections. In general, B cell-derived malignancies share a variety of immunological characteristics, such as leukopenia, T-cell exhaustion, and B cell dysfunction that, in part, contribute to the patients’ increased risk of infections [7, 9, 10]. As such, it stands to reason that patients with other B cell-derived malignancies than CLL would also demonstrate a sudden increase in the risk of infections shortly before diagnosis, possibly preceded by patterns indicating prolonged pre-diagnostic periods of immune dysfunction. Moreover, we previously argued that pre-diagnostic infections occurring shortly before diagnosis play, for all practical purposes, a similar causal role in terms of mortality as infections occurring between diagnosis and the start of treatment, as the precise timing of the lymphoma diagnosis can be construed as somewhat arbitrary [11]. We thus further expanded the analysis to include an investigation of the prognostic significance of pre-diagnostic infections in the context of other established predictors of survival.

Using data from Danish population-wide registers, we hypothesized that, like CLL, diffuse large B cell lymphoma (DLBCL), multiple myeloma (MM), follicular lymphoma (FL), marginal zone lymphoma (MZL), and lymphoplasmacytic lymphoma (LPL) would also demonstrate varying degrees of immunodeficiency, and thus higher rates of infections prior to diagnosis. Subsequently, for each of these malignancies, we assessed the clinical impact of recent pre-diagnostic infections on overall survival (OS) from the time of diagnosis in the context of other established international indices (IPIs).

## Methods

### Cohorts

Our investigation made use of two similar, but separate, data sources resulting in two cohorts: a *population cohort* and a *clinical cohort*, with overlapping time periods and patients. Cohort selection criteria are described in detail in the supplementary information (Supplementary Fig. 1, Supplementary Methods). Briefly, the *population* cohort was assembled using nationwide population- and cancer registers and allowed for matched case-control analyzes of pre-diagnostic use of antimicrobials [12, 13]. We identified all patients aged 18 and older diagnosed with a B cell-derived malignancy between June 1996 and Oct 2019 in the Danish Cancer Register. Each patient was matched with a minimum of five and up to 15 controls from the general population, matching on birth year and sex, such that the control was ensured to be alive and to not have been diagnosed with the same hematological malignancy at the time of diagnosis of their assigned patient. On the other hand, the *clinical* cohort was assembled from nationwide clinical quality registers providing information on IPIs and finer details on prognostic factors such as immunoglobulin heavy-chain variable (IGHV) status in CLL and cell of origin (COO) status in DLBCL. This enabled corresponding analyzes of pre-diagnostic use of antimicrobials stratified by prognostic factors. The clinical cohort was also used to investigate the impact of pre-diagnostic antimicrobials on OS after diagnosis in the context of known prognostic factors, specifically IPIs [14–19]. Notably, the clinical cohort comprised only patients and no population controls.

Throughout, each B cell-derived malignancy subtype was addressed in a separate analysis, subtypes being DLBCL, MM, FL, MZL (mæucosa-associated lymphoid tissue [MALT] lymphoma and nodal MZL [nMZL]), and LPL including Waldenström’s macroglobulinemia. Subtypes were classified using ICD-10 and ICD-O-3 codes (see supplementary – Cancer type groupings).

### Antimicrobial prescriptions

Data on antimicrobial prescriptions were retrieved from the Danish National Prescription Register from June 1996 through Oct 2019 and from Jan 2004 through Dec 2020 for the population cohort and clinical cohort, respectively (see Supplementary Methods) [20]. In Denmark, antimicrobials are not available over-the-counter. Even so, prescription data do not include antimicrobials administered in-hospital. We divided antimicrobial prescriptions into subgroups based on anatomical therapeutic chemical (ATC) codes as previously published [8]. Multiple antimicrobial prescriptions filled within 14 days of one-another were considered a single infectious event. This design was implemented across ATC-subgroups for the main analyzes and per ATC-subgroup for supplementary analyzes.

### Statistical Analysis

#### Prediagnostic antimicrobial prescriptions

We followed all individuals in the population cohort from June 1996 or the date of birth, whichever came later, until the diagnosis of B cell-derived malignancy. Controls were followed until the time of the diagnosis of their assigned case (i.e. pseudodiagnosis). Using Cox regression, we estimated hazard ratios (HR) for antimicrobial prescription in one-year intervals, comparing subjects who would later be diagnosed with CLL, DLBCL, MM, FL, MZL, and LPL, to their matched controls. In this analysis, we used age as the underlying time and stratified baseline hazards such that each case and their set of matched controls constituted a stratum. The regression was further adjusted for geographical region (time-dependent) as the strata were not defined to match on region, only on birth year and sex.

The clinical cohort was used for corresponding analyzes of the risk of prediagnostic antimicrobial use stratified by prognostic or morphological markers, i.e., disease-specific IPI, or IGHV- or COO status in individuals later developing CLL or DLBCL, respectively. In addition, these analyzes were adjusted for age, sex, calendar period at diagnosis, and geographical region at diagnosis.

#### Survival after diagnosis

The clinical cohort was used for Cox-regression to estimate HRs for all-cause mortality among patients, comparing subjects according to the number of antimicrobial prescriptions filled (0 vs 1 vs 2 vs >2) within one year prior to their diagnosis - henceforth referred to as *recent infections*. Survival analyzes were additionally adjusted for sex, age at diagnosis, calendar period at diagnosis, geographical region at diagnosis, and the corresponding IPI. In sensitivity analyzes, we examined whether the prognostic significance of prediagnostic antimicrobial use systematically varied according to sex or if the individual was diagnosed before or after age 50 years, which did not seem to be the case (results not shown).

Statistical analyzes were performed using R version 4.0.3 [21]. Register data were obtained through Danish Health Data Authority [22]. The study was approved by SSI QC and Compliance (j.nr. 21/00805) (population cohort) and National Ethics Committee 1804410 with Data Protection Agency P-2020-561 (clinical cohort).

## Results

The population cohort included 30,389 individuals diagnosed with a B cell-derived malignancy with a total observed time at risk for antimicrobial prescriptions of 355,372 person-years occurring before diagnosis, accumulating 213,649 antimicrobial prescriptions in that period. Controls comprised 435,846 individuals with a total observed time at risk of 5,238,485 person-years occurring before pseudodiagnosis, accumulating 2,748,300 antimicrobial prescriptions (Table 1).

**Table 1:** Descriptive characteristics of the population cohort

Variable	CLL	DLBCL	MM	FL	MZL	LPL
Cases, *n* (%)						
Female	3406 (40.2)	3361 (44.8)	3393 (44.5)	2041 (51.8)	588 (53.6)	659 (37.7)
Male	5073 (59.8)	4137 (55.2)	4230 (55.5)	1901 (48.2)	510 (46.4)	1090 (62.3)
Age at diagnosis, mean (SD)	70.6 (11.5)	66.7 (14.8)	69.7 (11.3)	62.7 (12.8)	66.3 (14.3)	71.7 (10.4)
Person years	95,715	87,165	90,366	47,100	14,585	20,441
Antimicrobial						
prescriptions, n (% ever)	33,421	32,915	33,640	16,117	5692	8328
J01A – Tetracyclines	1820 (9.2%)	1812 (9.8%)	1603 (9.2%)	1074 (10.3%)	212 (10.5%)	559 (10.5%)
J01C – Penicillins	33,421 (78.1%)	32,915 (78.8%)	33,640 (79.9%)	16,117 (77.5%)	5692 (86.2%)	8328 (80.1%)
J01E – Sulfonamides	5907 (22.4%)	5443 (22.4%)	5418 (24.2%)	2338 (21.5%)	987 (26.2%)	1299 (22.7%)
J01F – Macrolides	10219 (41.8%)	9259 (42.9%)	9719 (43.8%)	4792 (42.9%)	1882 (50.8%)	2525 (46.9%)
J01M – Quinolones	2263 (13.7%)	2238 (15%)	2477 (15.2%)	834 (12%)	367 (15.4%)	535 (15.8%)
J01X – Other antimicrobials	1731 (6.2%)	1669 (6.6%)	1456 (6.7%)	548 (4.6%)	225 (6.4%)	446 (7.3%)
J02A – Antimycotics	2234 (9.5%)	2354 (11%)	2067 (11.4%)	1356 (12.4%)	398 (13.2%)	637 (9.9%)
J05A – Antivirals	2161 (9.7%)	1702 (9.5%)	1713 (10.4%)	713 (7.8%)	388 (13.4%)	420 (11.1%)
P01A – Antiprotozoals	1376 (11.1%)	1340 (12.7%)	1336 (11.7%)	869 (14.2%)	307 (16.6%)	357 (13.1%)
P02C - Antihelminthics	243 (1.9%)	269 (2.3%)	257 (2.1%)	145 (2.4%)	73 (3.1%)	49 (1.9%)
Any	55,996 (86.1%)	53,740 (86.4%)	54, 395 (87.7%)	26 337 (85.8%)	9 492 (92%)	13 689 (88.5%)
Controls, *n* (%)						
Female	48,874 (40.6)	48,437 (45.0)	48,799 (44.9)	29,837 (51.8)	8638 (53.6)	9652 (38.0)
Male	71,516 (59.4)	59,129 (55.0)	59,940 (55.1)	27,792 (48.2)	7480 (46.4)	15,752 (62.0)
Age at pseudodiagnosis, mean (SD)	70.4 (11.4)	66.5 (14.7)	69.6 (11.2)	62.6 (12.7)	66.2 (14.2)	71.5 (10.3)
Person years	1,406,784	1,285,746	1,331,515	697,497	215,841	301,102
Antimicrobial						
Prescriptions, n (% ever)	84,446	75,474	79,716	37,509	13,430	18,068
J01A – Tetracyclines	24,037 (8%)	22,613 (8.4%)	23,388 (8.5%)	13,431 (9.2%)	3873 (9.5%)	5255 (8.4%)
J01C – Penicillins	441,739 (71.9%)	401,664 (73.3%)	421,789 (73.7%)	218,015 (74.5%)	69,185 (79%)	95,219 (71.6%)
J01E – Sulfonamides	84,446 (21%)	75,474 (21.4%)	79,716 (22.1%)	37,509 (21.2%)	13,430 (24.7%)	18,068 (21.4%)
J01F – Macrolides	123,611 (36%)	112,396 (36.8%)	119,106 (37.8%)	63,993 (38.8%)	19,784 (41.9%)	26,547 (36.6%)
J01M – Quinolones	29,387 (12.6%)	25,131 (12.3%)	27,315 (13%)	12,349 (11.6%)	4268 (14%)	6670 (13.6%)
J01X – Other antimicrobials	25,568 (6%)	21,226 (5.9%)	23,750 (6.2%)	9896 (5.3%)	3697 (6.8%)	5855 (6.3%)
J02A – Antimycotics	29,800 (9.1%)	29,733 (10.2%)	30,559 (10.2%)	20,245 (12%)	5845 (12.5%)	6672 (9.6%)
J05A – Antivirals	21,606 (7.4%)	19,916 (7.4%)	21,143 (7.8%)	12,348 (7.6%)	3538 (8.7%)	4619 (7.8%)

The clinical cohort included 18,560 subjects in total. Pre-diagnostic time at risk for antimicrobials totaled 174,752 person-years, during which 107,268 prescriptions of antimicrobials were filled (Supplementary Fig. 1). Post-diagnostic follow-up totaled 81,624 person-years with a median of 10.2 years (interquartile range: 6.4–13.2), during which 6697 (36.1%) subjects died (Table 2).

**Table 2:** Descriptive characteristics of the clinical cohort.

Variable	CLL	DLBCL	MM	FL	MZL	LPL
	*n* = 3342	*n* = 6361	*n* = 4422	*n* = 2268	*n* = 964	*n* = 1203
Sex, *n* (%)						
Female	1296 (38.8)	2798 (44.0)	1984 (44.9)	1162 (51.2)	521 (54.0)	456 (37.9)
Male	2046 (61.2)	3563 (56.0)	2438 (55.1)	1106 (48.8)	443 (46.0)	747 (62.1)
Age at diagnosis, mean (SD)	69.5 (10.7)	67.0 (13.7)	70.2 (11)	64.4 (12.0)	67.7 (13.8)	71.8 (9.9)
IPI*, n (%)						
Very low	-	-	1207** (27.3)	-	-	222 (18.5)
Low	1 740 (52.1)	468 (7.4)	368 (8.3)	376 (16.6)	237 (24.6)	348 (28.9)
Intermediate	941 (28.2)	3000 (47.2)	2151 (48.6)	1408 (62.1)	409 (42.4)	356 (29.6)
High	547 (16.4)	2893 (45.5)	696 (15.7)	484 (21.3)	318 (33.0)	194 (16.1)
Very high	114 (3.4)	-	-	-	-	83 (6.9)
Recent antimicrobials, n (%)						
0	1932 (57.8)	3208 (50.4)	2336 (52.8)	1362 (60.1)	513 (53.2)	635 (52.8)
1	822 (24.6)	1773 (27.9)	1117 (25.3)	546 (24.1)	232 (24.1)	311 (25.9)
2	325 (9.7)	763 (12.0)	522 (11.8)	212 (9.3)	103 (10.7)	130 (10.8)
>2	263 (7.9)	617 (9.7)	447 (10.1)	148 (6.5)	116 (12.0)	127 (10.6)
Prediagnostic follow up						
Antimicrobial prescriptions, n	19,789	34,312	27,117	11,159	6415	8476
Person years	34,110	55,387	43,986	19,546	9091	12,632
Post-diagnostic follow up						
Deaths, n (%)	804 (24.1)	2676 (42.1)	2094 (47.4)	535 (23.6)	269 (27.9)	319 (26.5)
Person years	15,417	28,582	14,400	13,551	4888	4786

^*^International prognostic indices and staging systems include CLL-IPI, R-IPI, R-ISS, FLIPI2, MALT-IPI, and rIPSSWM for CLL, DLBCL, MM, FL, MZL, and LPL, respectively. R-ISS stages I, II, and III correspond to low, intermediate, and high risk, respectively.

^**^Patients with SMM were classified as ‘Very low’ R-ISS.

### Prediagnostic use of antimicrobials

**Fig. 1:**
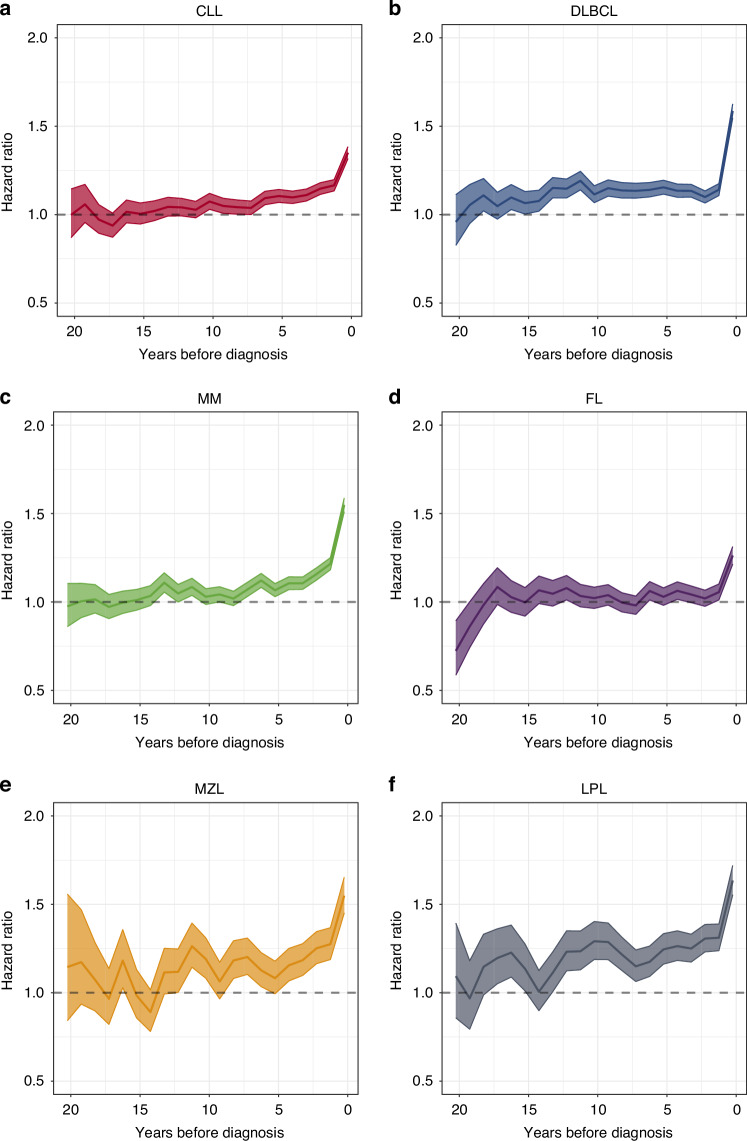
Patterns of antimicrobial use preceding diagnosis of B cell derived malignancy. **a** chronic lymphocytic leukemia (CLL), **b** diffuse large B cell lymphoma (DLBCL), **c** multiple myeloma (MM), **d** follicular lymphoma (FL), **f** marginal zone lymphoma (MZL), and lymphoplasmacytic lymphoma (LPL) including Waldenström macroglobulinaemia. Shown are hazard ratios with 95% confidence intervals for the use of any type of antimicrobial in cases relative to matched controls at distinct periods prior to diagnosis.

For each separate disease entity, patients demonstrated an increased risk of antimicrobial prescriptions in the year preceding diagnosis relative to their matched controls. Except for FL, we observed that the diagnosis of any B cell-derived malignancy was preceded by several years of increased use of antimicrobials as compared with matched controls. However, the timing and the offset of the accelerating rate of pre-diagnostic use of antimicrobials varied somewhat according to the specific disease entity (Fig. 1).

Among patients diagnosed with FL, the use of antimicrobials overlapped with that of their matched controls throughout all periods of observation until the year before diagnosis (Fig. 1d). By contrast, patients who developed CLL, MM, MZL, and LPL demonstrated a pre-diagnostic antimicrobial use that exceeded that of their matched controls at least a decade prior to diagnosis. This difference was noted to be increasing as the diagnosis approached, with the trend of increasing use accelerating at least five years prior to diagnosis (Fig. 1a, c, e, and f). Much like the other diseases, DLBCL demonstrated a marked increase in the use of antimicrobials in the last year prior to diagnosis, however, DLBCL was preceded by two decades of stable and elevated antimicrobial use as compared to their matched controls (Fig. 1b).

Subgroup analyzes of ATC groups were conducted using the population cohort (Supplementary Figs. 2–7). Penicillins were the most frequently used antimicrobials across all malignancies, followed by macrolides (Table 1).

Investigating the HRs of antimicrobial prescriptions for each ATC-subgroup as a function of time until diagnosis, we next highlighted some differences in trends between malignancies. For instance, future CLL patients demonstrated significantly increased HRs of macrolide prescriptions and antiviral prescriptions for more than a decade prior to diagnosis as compared to their controls (Supplementary Fig. 2). Future DLBCL patients demonstrated a consistent and stable increased risk for penicillin prescriptions starting more than a decade prior to their diagnosis. Likewise, the pattern of macrolide prescriptions in future DLBCL patients was similar to that of future CLL patients, with the HRs for macrolide prescriptions seeing a steady increase leading up to diagnosis (Supplementary Fig. 3). The same held true for future MM, MZL, and LPL patients (Supplementary Figs. 4, 6, and 7). For antimicrobials other than penicillin, the HRs for which were nominally elevated, we noted no significant trends in HRs for antimicrobial prescriptions for future FL patients when stratifying by antimicrobial subgroup (Supplementary Fig. 5).

Analyzes of the pre-diagnostic antimicrobial use stratified by IPI, IGHV status, and COO status did not demonstrate easily interpreted differences between strata (Supplementary Figs. 8–10).

### Survival after diagnosis

**Fig. 2:**
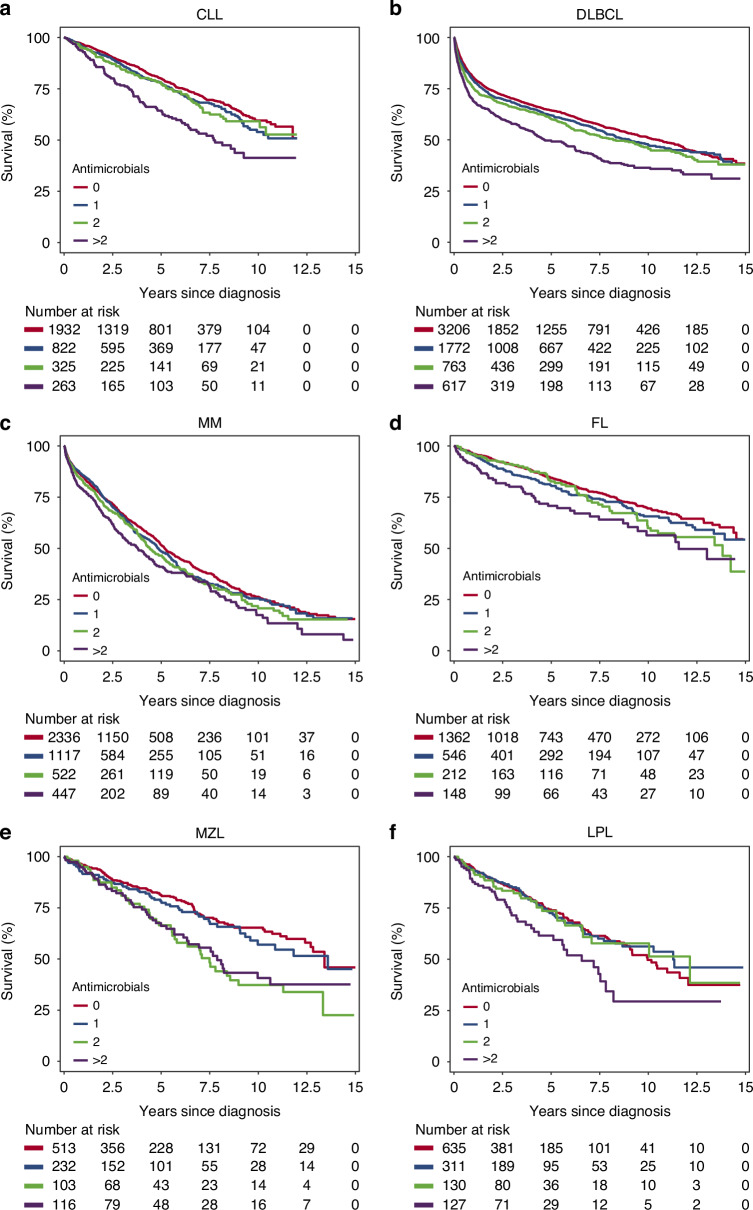
Prognostic implications of antimicrobial use preceding diagnosis B cell-derived malignancy. Shown are Kaplan–Meier curves for all-cause mortality after diagnosis, stratified by the total number of antimicrobial prescriptions within one year prior to diagnosis.

**Fig. 3:**
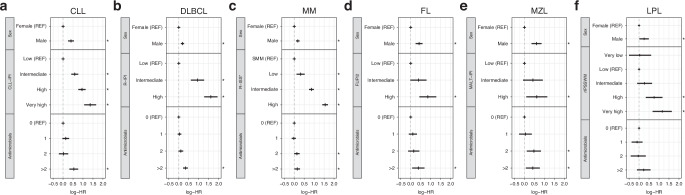
Effects of prognostic factors on all-cause mortality after diagnosis. Shown are hazard ratios obtained from separate Cox-regressions adjusted for age, calendar period and geographical region at diagnosis, with time since diagnosis as the underlying time scale.  Analyses were stratified by disease type: **a** chronic lymphocytic leukemia (CLL), **b** diffuse large B cell lymphoma (DLBCL), (**c**) multiple myeloma (MM), (**d** follicular lymphoma (FL), **e** marginal zone lymphoma (MZL), and (**f**) lymphoplasmacytic lymphoma including Waldenström macrogobulinaemia (LPL). * indicates *p* < 0.05.

Across all examined malignancies, we demonstrate shorter OS from the time of diagnosis with an increasing number of infections in the year preceding diagnosis (Fig. 2; *p* < 0.001). After adjusting for age at diagnosis, sex, calendar period of diagnosis, geographical region at diagnosis, and disease-specific IPI, having filled more than two antimicrobial prescriptions within one year before diagnosis continued to associate with significantly shorter OS as compared to having filled none for all malignancies examined (Fig. 3), except in the case of LPL (Fig. 3f). The association between antimicrobial prescriptions and OS was most pronounced in CLL, DLBCL, MZL, and LPL. (Fig. 2) At the same time, the disease-specific prognostic scores maintained their independent prognostic value for all examined malignancies. (Fig. 3, Supplementary Table 3).

## Discussion

We used antimicrobial prescriptions as a proxy for infections and compared individuals who would later develop B cell-derived malignancies to matched controls for extensive periods of time before diagnosis. In doing so, we showed that except for FL the risk of infections had been increased several years prior to diagnosis of all the examined malignancies. We also showed that for all disease types, the risk of infections increased markedly in the year just prior to diagnosis, likely a consequence of undiagnosed malignancy. We next compared overall survival among subjects with cancer stratified by the number of recent infections. In survival analyzes using comprehensive prognostic information, we demonstrated that treatment for more than two recent infections was associated with shorter OS, independent of age, sex, calendar period, geographical region, and IPI.

### Prediagnostic infections

While ours is not the first investigation to report an increased risk of infections before diagnosis of B cell-derived malignancy [8, 23, 24], we were able to expand on previous observations with detailed analyzes of temporal variations within and between the different malignancies. Disregarding the last year before diagnosis, three different patterns of pre-diagnostic infections could be discerned for the examined B cell-derived malignancies, suggestive of different pathways of pathogenesis. The first pattern was observed in individuals who were later diagnosed with CLL, MM, LPL, and MZL, such that the risk of infections had gradually increased over the span of a decade or more (Fig. 1). This pattern indicated a continuous deterioration of immune competence in conjunction with – and possibly because of – the development of these malignancies. For CLL, MM, and LPL this assumption aligns with previous observations as disease precursors such as monoclonal B-lymphocytosis and monoclonal gammopathy of undetermined significance (both IgM and non-IgM) can be demonstrated decades prior to overt malignancy have been associated with an increased risk of infections [9, 25–27]. As previously discussed for CLL [8], we cannot from these analyzes determine if recurrent infections play a causal role in the development of these malignancies. The increased risk of prediagnostic infections could be causally unrelated to the pathogenesis and merely reflect an underlying immune deficiency, or the infections could promote the development of indolent precursors and/or to the precursors’ continued transformation into malignant cells. In the latter case, the recurrent infections and the induced immune dysfunction would act in synergy to accelerate pathogenesis beyond their individual effects.

MZL has been associated with specific chronic infections and with autoimmune conditions. The underlying biological mechanism behind these associations is one involving persistent antigenic drive resulting in poly-, oligo- and ultimately monoclonal B cell expansion [28]. Interpreting the observed prolonged period of increased use of antimicrobials before diagnosis as being due to underlying immune dysfunction is compatible with this understanding of MZL pathogenesis. Meanwhile, we speculate that the accelerating use of antimicrobials starting around 5 years prior to diagnosis may be an early manifestation of lymphoma-induced immune dysfunction [29].

A second pattern of pre-diagnostic infections was observed in individuals who developed DLBCL. This group of patients demonstrated a 10% increased risk of infections for up to 15 years prior to the typical accelerating rate of antimicrobial use heralding diagnosis of all examined lymphomas (Fig. 1b). Profound immunodeficiencies as seen with, e.g., AIDS or organ transplantation as well as immunologic conditions like autoimmune diseases have both been linked with the risk of DLBCL [30–34]. Also, causal associations have been established between certain infections and DLBCL, e.g., with EBV especially in the setting of overt immune deficiency [1, 35].

Again, we cannot from our investigation determine the precise mechanisms underlying the observed pattern of pre-diagnostic infections, i.e., whether and to what extent immunodeficiency facilitates infections’ direct and indirect contributions to lymphomagenesis. We note, however, that in a previous investigation elevated levels of serological markers of immune activation could be demonstrated more than 7.5 years before DLBCL diagnosis [29]. We therefore speculate that the observed association between infections and DLBCL reflects the scenario when immunodeficiency predisposes to DLBCL via failure to eradicate malignant cells, failure to curb oncogenic infections, and through continuous immune stimulation caused by recurrent infections.

The third pattern of pre-diagnostic infections was observed for individuals who developed FL who, unlike patients with any of the other studied B cell derived malignancies, had similar use of antimicrobials as their controls until shortly before the diagnosis of malignancy. FL etiology remains poorly understood and the few risk factors for FL that have been suggested would account only for a small proportion of cases [36]. Our study indicates that neither risk factors for FL nor FL precursor states appear to manifest as an increased risk of infections and that FL stands out from other B cell malignancies insofar as immune deficiency is not a hallmark of FL risk. Previous studies have also found that immune deficiency does not inherently elevate the risk of developing FL, corroborating our results [37].

We hypothesized that aggressive disease, reflected by a high-risk IPI score and non-GCB- and unmutated IGHV status in DLBCL and CLL, respectively, would be indicative of rapid tumor growth and subsequently lower historical prediagnostic rates of use of antimicrobials (i.e. length time bias) [38, 39]. However, with the exception of CLL-IPI high and very high risk compared to CLL-IPI low risk, we did not observe any difference in the prediagnostic risk of infections according to IPI, COO- or IGHV status (Supplementary Figs. 8–10). These analyzes were statistically well-powered and suggest a similar increased risk of prediagnostic infections in individuals who are later diagnosed with aggressive and more indolent phenotype CLL and DLBCL.

To summarize, dependent on disease subtype, individuals diagnosed with B cell-derived malignancies demonstrated an increased risk of infections up to 15 years prior to diagnosis, which accelerated markedly the year just prior to diagnosis. Different patterns of antimicrobial use, superimposed, suggest distinct immunological phenomena at play, one prolonged and one acute.

Examination of the biological mechanisms underlying the former phenomenon may shed further light on lymphoma pathogenesis and pave the way for future preventative strategies; the latter may have more immediate clinical implications for prognostication, discussed forthwith.

### Survival after diagnosis

B cell-derived malignancies are subject to significant diagnostic delay, with some studies reporting a delay of three to five months even for symptomatic patients [40]. Undiagnosed lymphoma is most likely also the explanation for the sudden increased risk of infections in the year prior to diagnosis. As infections after diagnosis of B cell-derived malignancy are associated with increased mortality [41, 42], we expected patients with multiple recent infections prior to diagnosis to also demonstrate shorter OS after diagnosis compared to patients without. Indeed, univariate analyzes demonstrated that having more than two recent infections associated with shorter OS after diagnosis (Fig. 2), and remained predictive of significantly shorter OS in analyzes adjusted for other studied prognostic factors (Fig. 3). The effect of recent infections on mortality was particularly pronounced in patients with DLBCL and CLL, and to a lesser extent in FL, MZL, and LPL, whereas recent infections barely affected survival after MM diagnosis. Interpreting the number of recent infections as a proxy for level of immunodeficiency suggests that the mechanisms are a combination of increased rates of infectious death and treatment constraints.

Although a leading cause of death in patients with B cell-derived malignancies, neither infections nor measures of immunodeficiency are included in current IPIs and staging systems [14–19]. If our findings are validated in an independent cohort, we suggest that more than two recent infections prior to diagnosis of B cell-derived malignancy are indicative of marked immunodeficiency and could be considered for inclusion in revised IPIs to refine gold standard prognostication.

### Strengths and Limitations

We performed a population-based epidemiological investigation of 30 389 and 18 560 patients in the population cohort and the clinical cohort, respectively, with near-complete information on prescriptions and follow-up. For the population cohort, we were able to match each patient with a minimum of five and up to 15 controls. All survival analyzes were performed using a clinical cohort with information on prognostic factors such as IPI available for all patients [43–46]. Further, all patients in the clinical cohort had at least one full year of recorded prescription history prior to diagnosis.

Information on specific pathogens, such as H. pylori, implicated in the lymphomagenesis of mucosa-associated lymphoid tissue (MALT) lymphomas was not available [1]. Interestingly, the use of both penicillin and macrolides were increased years prior to MZL diagnosis and demonstrated the highest absolute risk of macrolide use across all malignancies investigated, suggesting that eradication therapy could indeed interfere with our observations (Supplementary Fig. 6) [47]. Our findings regarding antiviral treatment should be interpreted with caution. On the one hand, most infectious viral syndromes are treated only symptomatically, while on the other hand they may be more commonly treated in individuals at risk of the infection to run a severe course, e.g., because of impaired immune function (Supplementary Figs. 2–7 ‘Antivirals’). These mechanisms are likely to both influence the observed associations. Further, B-symptoms related to malignancy may commonly be mistaken for routine infections [48], and some newly diagnosed lymphoma and MM patients demonstrate elevated C-reactive protein levels [49, 50]. Thus some antimicrobial prescriptions may represent early malignancy rather than true infections. Likewise, others have suggested that the risk of being diagnosed with a B cell-derived malignancy is presumably higher among heavy antimicrobial users during routine follow-up and visits related to comorbidities [8]. When analyzing pre-diagnostic antimicrobial use, we did not adjust for comorbidities as the diagnosis of a (future) malignancy was implemented as an exposure, and subsequently used to assess differences in rates of antimicrobial use in the years preceding (pseudo)diagnosis between future patients and controls. Comorbidities, in this context, would therefore serve as a mediator of the effect of future malignancy on preceding rates of antimicrobial use. Therefore, any adjustment for comorbidities would serve to obfuscate the differences in rates of antimicrobial use observed among future patients and controls. In a similar vein, regarding analyzes of survival after diagnosis, we have previously shown that comorbidities in patients with CLL are associated with a higher risk of dying from infections [5]. We did not adjust for comorbidities in our analyzes of survival after diagnosis, as these were designed only to shed light on the independent effects of recent infections when combined with established prognostic factors such as IPIs, age, and sex. Specific comorbidities could be subject for further investigation in the context of infections and B cell-derived malignancies [51]. Lastly, the patients in the population and clinical cohorts overlap to a large degree, but due to mandatory pseudonymization being carried out by two separate institutions, we could not determine to what extent.

## Conclusion

The risk of infections was significantly increased up to 15 years prior to diagnosis of B cell-derived malignancies and accelerating just prior to diagnosis, and only in the case of FL was the former pattern not observed. Interpreting the findings as resulting from two superimposed patterns of antimicrobial use, one prolonged and one acute aligns with previous observations of long-lasting immunodeficiency preceding sudden cancer emergence. Antimicrobial use and disease aggressiveness as determined by established IPIs were not mutually associated. Further examination of the biological mechanisms underlying these phenomena may shed further light on lymphoma pathogenesis and pave the way for future preventative strategies. Patients treated for more than two recent infections before diagnosis of B cell-derived malignancy demonstrated shorter OS even after adjusting for other known prognostic factors. Thus, the number of recent infections may constitute an independent prognostic tool and could be considered in combination with current IPIs and staging systems to predict patient survival more accurately.

## Supplementary information

Supplementary

## Data Availability

According to Danish legislation, the authors are unable to share data; however, researchers can be granted access to work with the data within the approved project on a collaborative basis based on individual assessment by the authors. All data can be acquired through the Danish Clinical Quality Program and the Danish Health Data Authority.
