# Supplementary material for: Infections and their prognostic significance before diagnosis of chronic lymphocytic leukemia, non-Hodgkin lymphoma, or multiple myeloma
Source: Br J Cancer. 2024 Aug 22;131(7):1186–94. doi: 10.1038/s41416-024-02816-2 (PMC11442662; doi:10.1038/s41416-024-02816-2)
Supplement: Supplementary file 1 — Supplementary [file 41416_2024_2816_MOESM1_ESM.pdf]

Supplementary

Table of content

Methods 3
Registers 3
Danish Civil Registration Systems 3
Danish National Patient Register 3
Danish National Prescription Register 3
Danish Cancer Register 3
Danish Pathology Register 3
Danish National CLL Registry 4
Danish Myeloma Register 4
Danish Lymphoma Register 4
Antimicrobial Prescriptions 4
Cohorts 5
Population cohort 5
Clinical cohort 5
Figures 7
supplemental Figure 1 – Consort diagram of the clinical cohort. 7
supplemental Figure 2 – Subgroup analyses of prediagnostic antimicrobial use in patients with chronic lymphocytic leukemia (CLL) relative to matched controls. 8
supplemental Figure 3 – Subgroup analyses of prediagnostic antimicrobial use in patients with diffuse large B-cell lymphoma (DLBCL) relative to matched controls. 9
supplemental Figure 4 - Subgroup analyses of prediagnostic antimicrobial use in patients with multiple myeloma (MM) relative to matched controls. 10
supplemental Figure 5 - Subgroup analyses of prediagnostic antimicrobial use in patients with follicular lymphoma (FL) relative to matched controls. 11
supplemental Figure 6 - Subgroup analyses of prediagnostic antimicrobial use in patients with marginal zone lymphoma (MZL) relative to matched controls. 12
supplemental Figure 7 - Subgroup analyses of prediagnostic antimicrobial use in patients with lymphoplasmacytic lymphoma (LPL) relative to matched controls. 13
supplemental Figure 8 – Prediagnostic antimicrobial prescriptions stratified by international prognostic indices. 14
supplemental Figure 9 – Prediagnostic prescriptions stratified by IGHV status in patients with chronic lymphocytic leukemia. 15

supplemental Figure 10 – Prediagnostic antimicrobial use stratified by COO-status in patients with diffuse large B-cell lymphoma (DLBCL). 16

Tables 17

supplemental Table 1: Defining SMM from the Danish Myeloma Database 17

supplemental Table 2 – International prognostic indices. 18

Methods

Registers

Danish Civil Registration Systems – Individuals living in Denmark are given a unique personal identification number in the Danish Civil Registration System (CRS). This register continuously monitors vital statistics of all residents in Denmark and contains their sex, date of birth, date of death, as well as their current residence and past relocation history.

Danish National Patient Register - The national patient register contains information on virtually all non-psychiatric hospital admissions in Denmark. Each hospital admission initiates a record, which includes the patient's personal identification number, admission and discharge dates, a primary discharge diagnosis, as well as supplementary diagnoses coded in accordance with ICD-8 from 1977 to 1993, and ICD-10 from 1994 and onwards.

Danish National Prescription Register - The Danish National Prescription Register contains individual-level information on all prescription drug purchases for the entire population of Denmark since 1994[3]. This includes the individuals' personal identification number, product name, manufacturer of the dispensed drug, and its Anatomical Therapeutic Chemical (ATC) classification. Notably, in the period from 1994 to 1997, prescriptions intended for children were often written using one of their parent's personal identification number.

Danish Cancer Register - The Danish Cancer Register contains records of incidence of cancer in the Danish population since 1943 with a high degree of completeness. As of 1978, the register also provides ICD-O-3 codes, including morphology, topography, and behavior of each diagnosis.

Danish Pathology Register – Since 1990 all departments of pathology in Denmark have used electronic recording of the diagnostic statements and the diagnoses were coded after a Danish version of the Systematized Nomenclature of Medicine (SNOMED). The database is mandatory by law with near complete coverage of all recorded material obtained within Danish hospitals.

Danish National CLL Registry – The National Danish CLL Register includes all patients diagnosed with CLL in Denmark from January 2008 onward. All Danish haematological centers participate in the registry and are all obliged to collect quality data.

Danish Lymphoma Register – Danish Lymphoma Register – or LYFO database – includes all newly diagnosed lymphomas in Denmark since 2000, although we were allowed access from records from January 2005 onward. All Danish haematological centers participate in the registry and are all obliged to collect quality data.

Danish Myeloma Register – The Danish Myeloma Register includes all newly diagnosed patients with MM, SMM, solitary plasmacytomas, and plasma cell leukemia in Denmark from January 2005 onward. All Danish haematological centers participate in the registry and are all obliged to collect quality data.

Antimicrobial Prescriptions – We extracted data on the use of tetracyclines (ATC: J01A-), penicillins (J01C-), sulfonamides (J01E-), macrolides (J01F-), quinolones (J01M-), other antibacterials (J01X-), antimycotics (J02A-), antivirals (J05A-), antiprotozoals (P01A), and antihelminthics (P02C-).

Cohorts

Population cohort - We defined a population-wide cohort of cases and matched controls identified among all Danish residents, born in Denmark, to conduct our analyses of prediagnostic antimicrobial prescriptions. A maximum of 15 controls were sampled for each case, matching on sex and year of birth. All controls were alive and cancer-free (all types) at the time of diagnosis of the index patient. Cases with less than five matched controls were excluded. Data on lymphoma diagnoses in the period from Jan 1st, 1978 to Dec 31st, 2017 were retrieved from the Danish Cancer Register. Based on a unique personal identification numbers from the Danish Civil Registration System, we were able to link information on antimicrobial prescriptions from the Danish Prescription Register to information on vital status and residential status. As such, the final population-based cohort comprised all individuals diagnosed with B-cell derived malignancies sometime between Jan 1st, 1978 and Dec 31st, 2017 as cases along with matched controls of at least five and up to 15 controls per case. Controls were allocated a pseudo-diagnosis date corresponding to the date at which their assigned case was diagnosed.

Clinical cohort - From the Danish Clinical Quality Program - National Clinical Registries, we identified all patients diagnosed with CLL since Jan 1st, 2008 from the Danish CLL Register, small lymphocytic lymphoma (SLL), LPL, MZL (MALT/nMZL), FL or DLBCL since Jan 1st, 2005 from the Danish Lymphoma Register and all patients with SMM or MM since Jan 1st, 2005 from the Danish Myeloma Register, until Jan 1st, 2020. Patients diagnosed with more than one B-cell derived malignancy were classified according to the first occurring diagnosis (supplemental Figure 1).

CLL and SLL were grouped as they are considered to be manifestations of the same disease entity. WM is characterized as bone marrow infiltration of LPL with detectable IgM-protein in serum. LPL and WM were grouped in the Danish Lymphoma Register as LPL and remained so for our analyses. Patients with MM were categorized as having SMM based on the absence of myeloma defining events and treatment within three months of diagnosis, as per Danish guidelines (supplemental Table 1). Patients with aggressive phenotype FL grade 3 having received R-CHOP (rituximab plus cyclophosphamide, doxorubicin, oncovin and prednisone)-like regimens (excluding R-CVP [rituximab plus cyclophosphamide, oncovin and prednisone])

within three months of diagnosis were categorized as FL grade 3b and grouped together with patients with DLBCL. We calculated prognostic indices including CLL-IPI, R-IPI, R-ISS, FLIPI2, MALT-IPI, and rIPSSWM for patients with CLL, DLBCL, MM, FL, MZL, and LPL, respectively (supplemental Table 2). Patients without complete prognostic scores were excluded from the survival analyses given the international prognostic indices' evident correlation with survival.

To investigate how future IGHV- and COO-status at time of CLL and DLBCL diagnoses, respectively, affect the prediagnostic risk of infections, we used SNOMED codes from the Danish Pathology Register. Specifically, 'ÆF5221' and 'ÆF5222' for GCB and non-GCB, respectively, and 'FE13N7' and 'FE13N8' for mutated IGHV and unmutated IGHV, respectively. As IGHV- and COO-status maintain its status throughout the course of disease, no bias should be introduced when predicting based on future status.

Cancer type groupings

Malignancy subtypes were identified in the population cohort using ICD-10 and ICD-O-3 codes as follows:

**Subtype**	**Definition**
CLL	ICD10 C91.1, C91.3 & C91.6.
DLBCL	ICD-O-3 96803
MM	ICD10 C90.0 – C90.3
FL	ICD-O-3 96903, 96913, 96953, & 96983
MZL	ICD-O-3 96993
LPL	ICD-O-3 96713 & 97613

Figures

supplemental Figure 1 – Consort diagram of the clinical cohort. *Patients with more than one malignancy were classified according to the first occuring diagnosis and censored hereafter

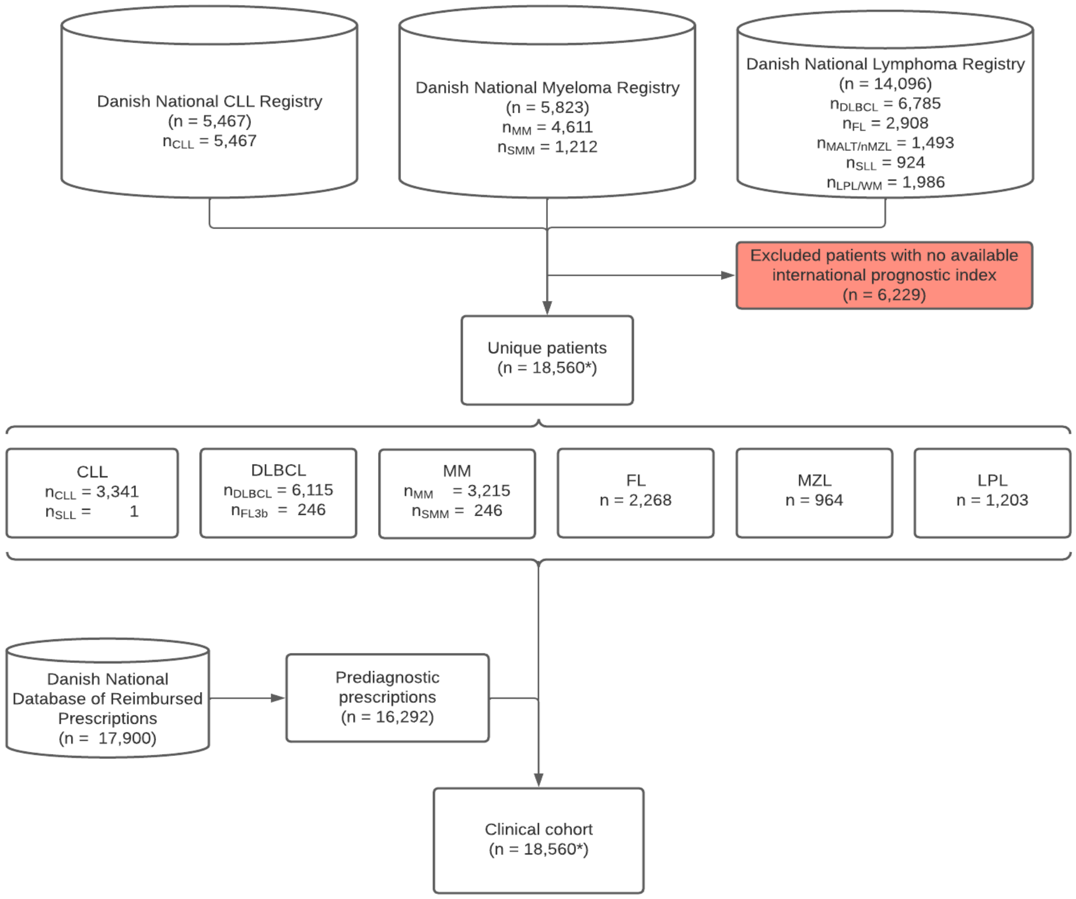

supplemental Figure 2 – Subgroup analyses of prediagnostic antimicrobial use in patients with chronic lymphocytic leukemia (CLL) relative to matched controls. *Hazard ratios with 95% confidence intervals for use of specific types of antimicrobials in CLL patients relative to matched controls (dashed line), stratified by time until diagnosis.*

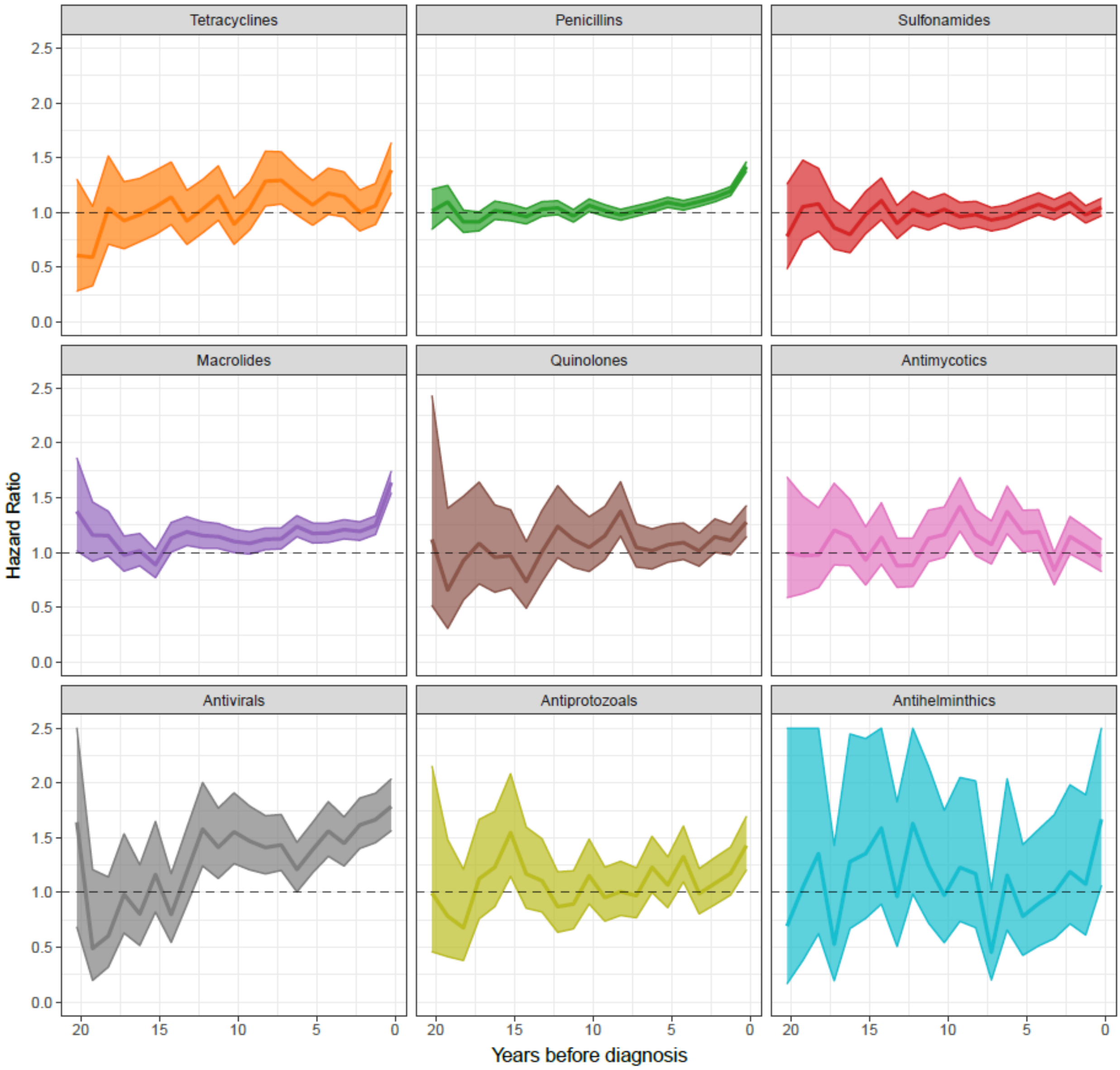

supplemental Figure 3 – Subgroup analyses of prediagnostic antimicrobial use in patients with diffuse large B-cell lymphoma (DLBCL) relative to matched controls. Hazard ratios with 95% confidence intervals for use of specific types of antimicrobials in DLBCL patients relative to matched controls (dashed line), stratified by time until diagnosis.

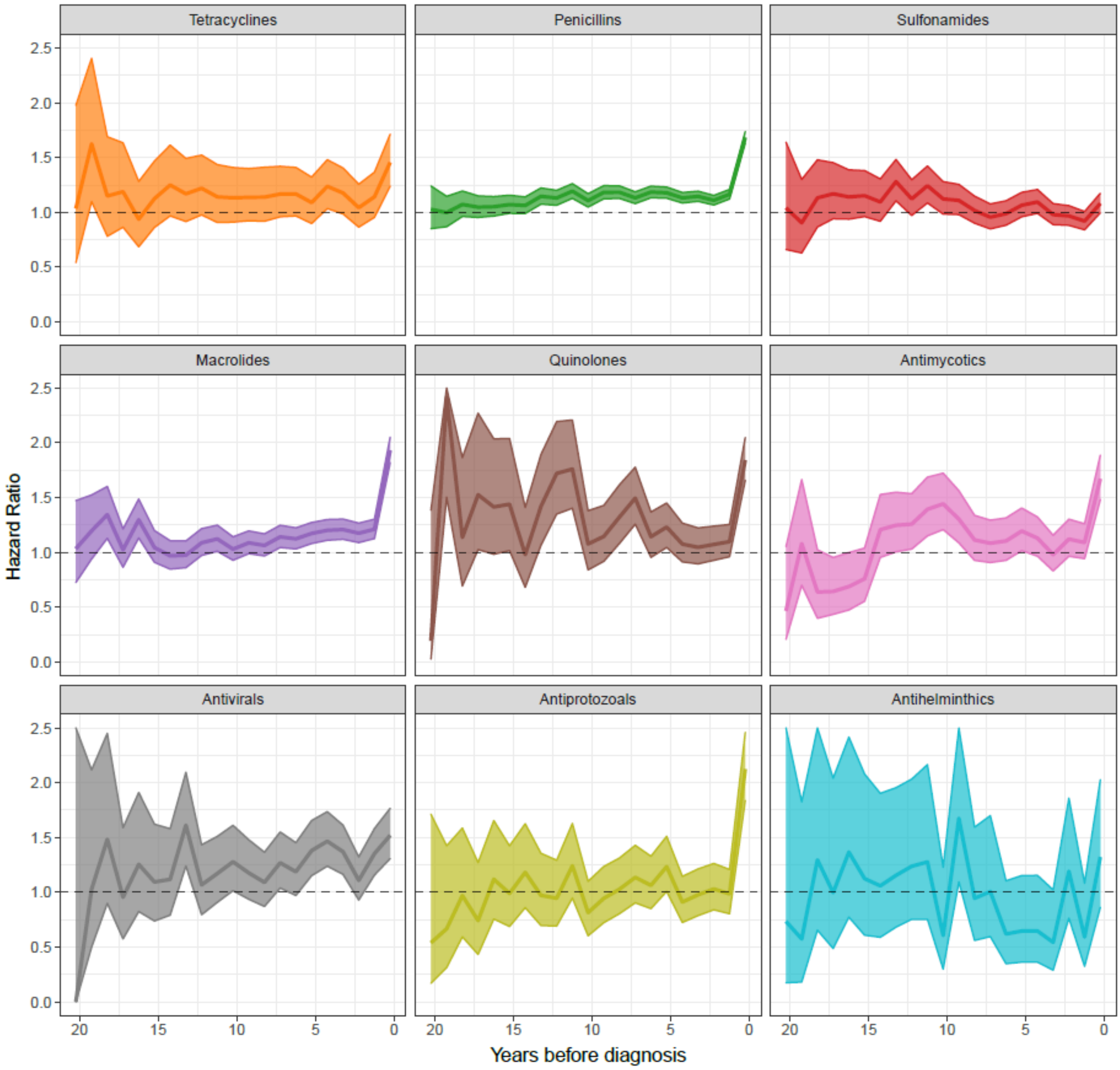

supplemental Figure 4 - Subgroup analyses of prediagnostic antimicrobial use in patients with multiple myeloma (MM) relative to matched controls. Hazard ratios with 95% confidence intervals for use of specific types of antimicrobials in MM patients relative to matched controls (dashed line), stratified by time until diagnosis.

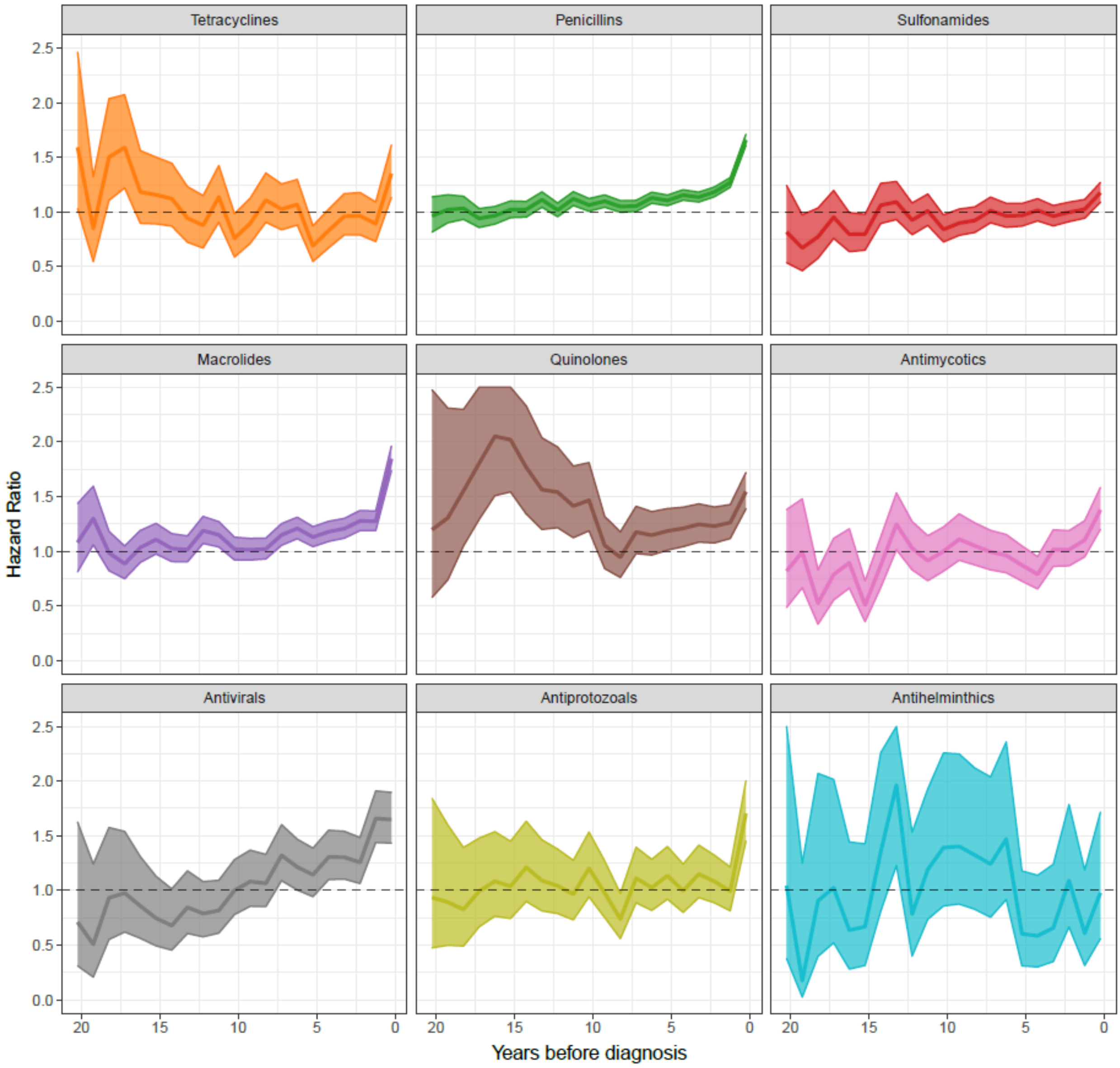

supplemental Figure 5 - Subgroup analyses of prediagnostic antimicrobial use in patients with follicular lymphoma (FL) relative to matched controls. Hazard ratios with 95% confidence intervals for use of specific types of antimicrobials in FL patients relative to matched controls (dashed line), stratified by time until diagnosis.

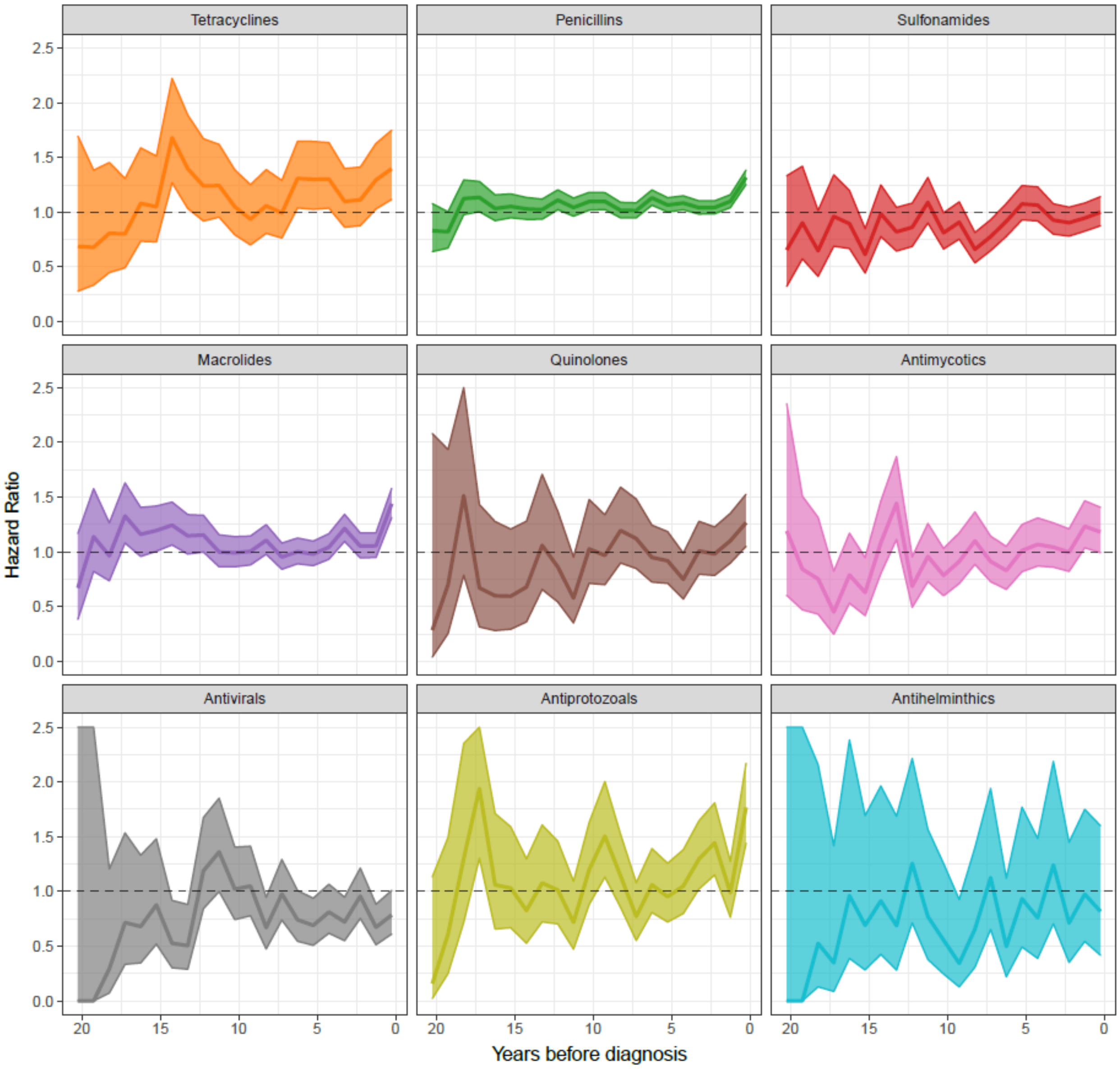

supplemental Figure 6 - Subgroup analyses of prediagnostic antimicrobial use in patients with marginal zone lymphoma (MZL) relative to matched controls. Hazard ratios with 95% confidence intervals for use of specific types of antimicrobials in MZL patients relative to matched controls (dashed line), stratified by time until diagnosis.

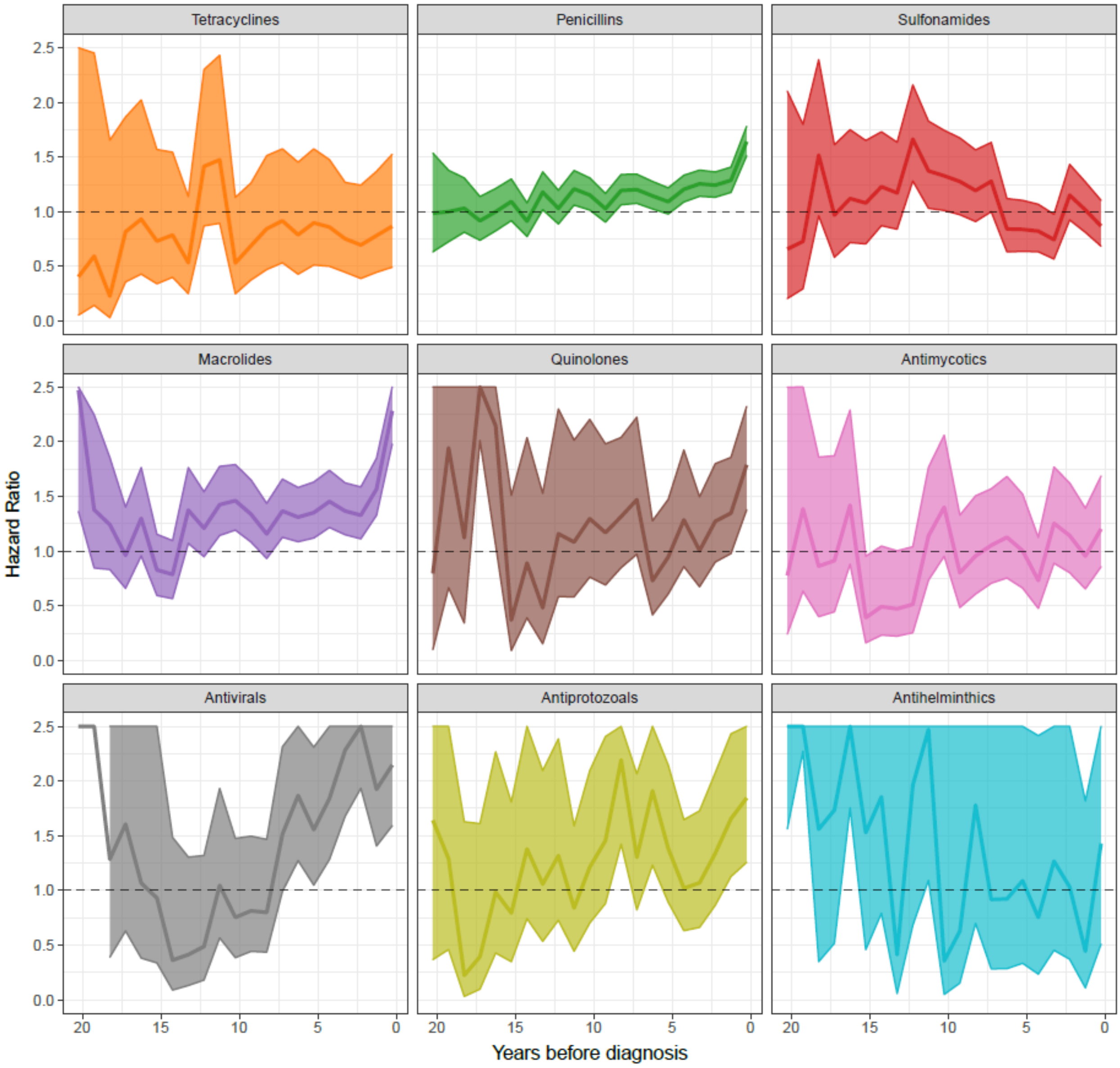

supplemental Figure 7 - Subgroup analyses of prediagnostic antimicrobial use in patients with lymphoplasmacytic lymphoma (LPL) relative to matched controls. Hazard ratios with 95% confidence intervals for use of specific types of antimicrobials in LPL patients relative to matched controls (dashed line), stratified by time until diagnosis.

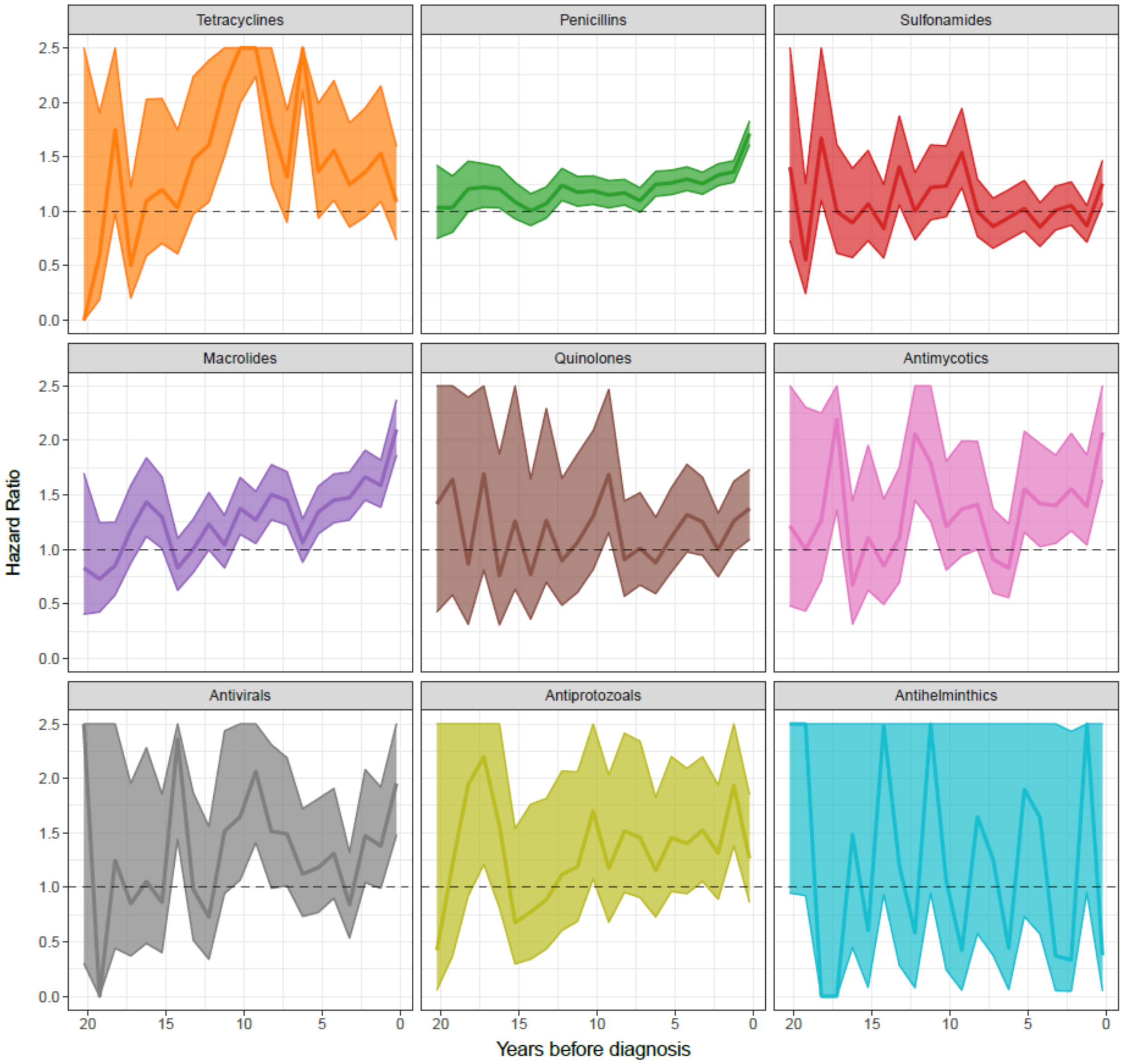

supplemental Figure 8 – Prediagnostic antimicrobial prescriptions stratified by international prognostic indices. ***CLL*** *– Hazard ratios with 95% confidence intervals stratified by time until diagnosis for antimicrobial prescriptions (HRs) in CLL patients with intermediate (blue) and high (red) CLL-IPI versus patients with low CLL-IPI (dashed line);* ***DLBCL*** *- HRs for antimicrobial prescriptions in DLBCL patients with intermediate (blue) and high (red) R-IPI versus patients with low R-IPI (dashed line);* ***MM*** *- HRs for antimicrobial prescriptions in MM patients with I (low, green), II (intermediate, blue), and III (high, red) RISS versus patients with SMM (dashed line);* ***FL*** *- HRs for antimicrobial prescriptions in FL patients with intermediate (blue) and high (red) FLIPI2 versus patients with low FLIPI2 (dashed line);* ***MZL*** *- HRs for antimicrobial prescriptions in MALT/nMZL patients with intermediate (blue) and high (red) MALT-IPI versus patients with low MALT-IPI (dashed line); and* ***LPL*** *- HRs for antimicrobial prescriptions in LPL patients with intermediate (blue) and high (red) rIPSSWM versus patients with low rIPSSWM (dashed line).*

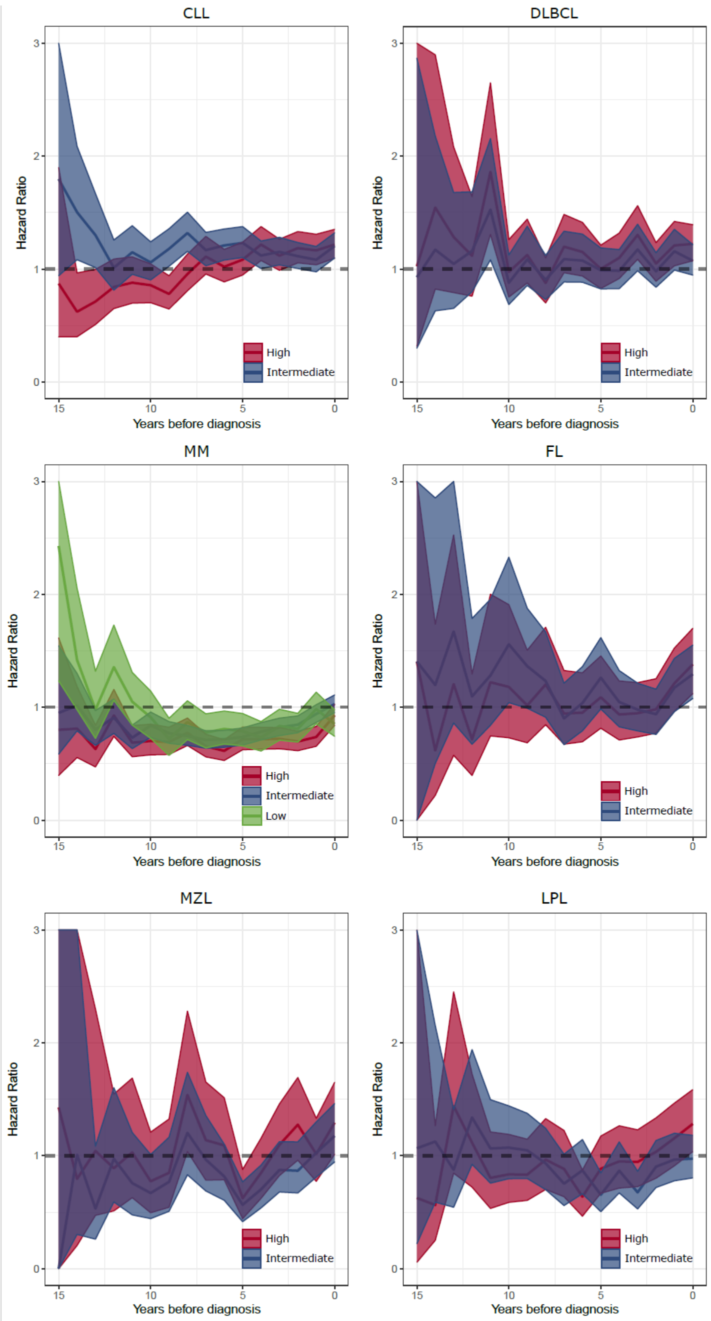

supplemental Figure 9 – Prediagnostic prescriptions stratified by IGHV status in patients with chronic lymphocytic leukemia (CLL). Hazard ratios with 95% confidence intervals for use of any type of antimicrobials in CLL, unmutated IGHV (red) versus CLL patients with mutated IGHV (dashed line), stratified by time until diagnosis.

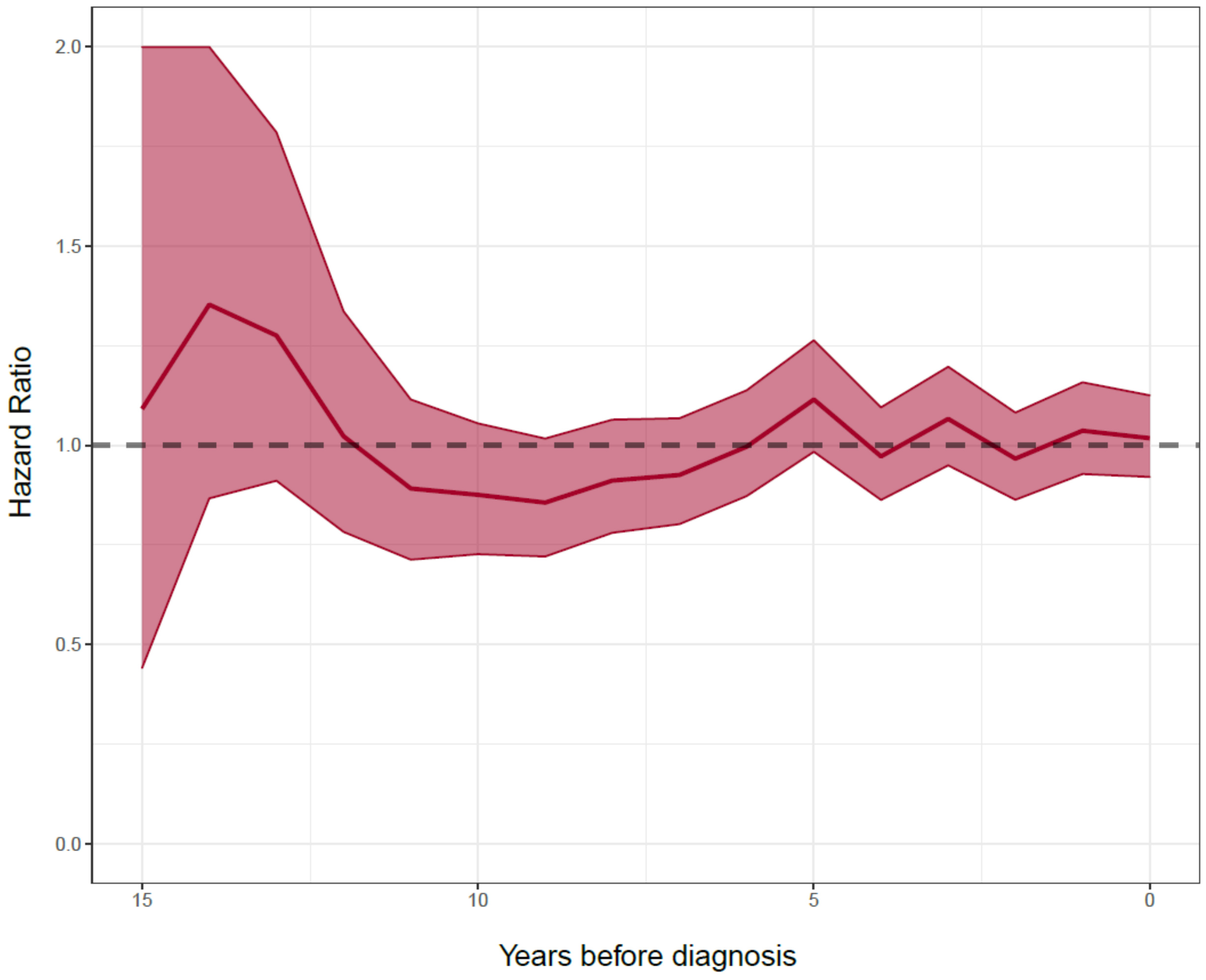

supplemental Figure 10 – Prediagnostic antimicrobial use stratified by cell of origin (COO)-status in patients with diffuse large B-cell lymphoma (DLBCL). Hazard ratios with 95% confidence intervals for use of any type of antimicrobials in DLBCL patients, COO non-GCB (red) versus DLBCL patients with COO GCB (dashed line), stratified by time until diagnosis.

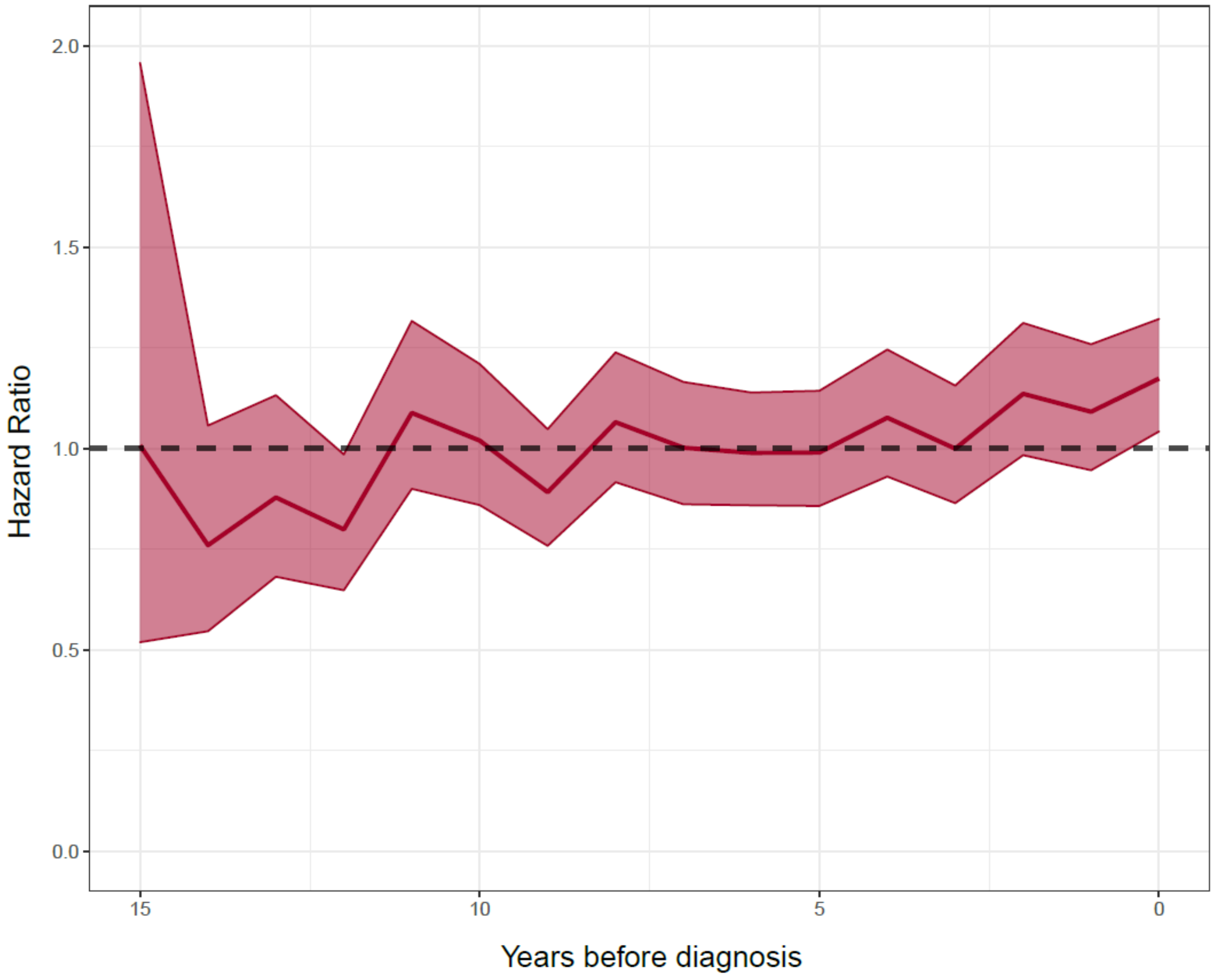

Tables

supplemental Table 1: Defining SMM from the Danish Myeloma Database

Criteria	Points	MM subtype
P-Ca^{2+} 1.4 mM	+ 1	0 points: SMM
P-Creatinine > 177 µM	+ 1	
Hemoglobin < 10 g/dL	+ 1	
Myeloma bone lesion	+ 1	> 0 points: MM
Treatment < 90 days	+ 1	
Dead < 90 days	+1	

CRAB in accordance with Rajkumar, American Journal of Hematology, 2022. Newer myeloma defining events (MDE) were not generally recommended in Denmark during the study follow-up.

Supplemental Table 2 – International prognostic indices. ISS, R-IPI and FLIPI2 were provided in the registers.

IPI	Criteria	Points	Score	IPI category
CLL-IPI	Age > 65 years	+ 1	0-1	Low
	Binet B/C	+ 1	2-3	Intermediate
	ß2 microglobulin > 4 mg/L***	+ 2	4-6	High
	IGHV unmutated*	+ 2	7-10	Very high
	del(17p) and/or *TP53* mutation	+ 4		
R-ISS	ISS 1 with LDH < 205 U/L and no FISH abbreviations**			I: Low
	ISS 1 with LDH > 205 U/L or FISH abbreviations**			
	ISS 2			II: Intermediate
	ISS = 3 with LDH < 205 and no FISH abbreviations**			
	ISS = 3 with LDH > 205 or FISH abbreviations**			III: High
MALT-IPI	Age ≥ 70 years	**+ 1**	**0**	Low
	Elevated LDH	+ 1	1	Intermediate
	Ann Arbor stage > 2	+ 1	2-3	High
rIPSSWM	65-75 years	+ 1	0	Very low
	Age > 75 years	+ 2	1	Low
	ß2 microglobulin > 4 mg/L	+ 1	2	Intermediate
	LDH > 250 U/L	+ 1	3	High
	Albumin < 35 g/L	+ 1	4 - 6	Very high

*If IGHV was not available in the CLL registry, IGHV status was provided by the Danish pathology registry. **t(4;14), t(14;16) or del(17p) detected by FISH. *** Modified using B2M > 4.0 mg/L as previously validated (da Cunha-Bang et al. Blood. 2016)

Supplementary Table 3 - Results underlying Figure 3

Subtype	Variable Category	Value	Log HR (95% CI)	HR (95% CI)	P-value
CLL	Sex	Female (Ref)	-	-	-
		Male	0.17 (0.11 - 0.23)	1.48 (1.28 - 1.72)	1.6.E-07
	CLL-IPI	Low (Ref)	-	-	-
		Intermediate	0.25 (0.18 - 0.33)	1.80 (1.51 - 2.14)	5.0.E-11
		High	0.41 (0.33 - 0.49)	2.56 (2.13 - 3.07)	6.5.E-24
		Very high	0.59 (0.46 - 0.72)	3.89 (2.89 - 5.25)	3.8.E-19
	Recent Antimicrobials	0	-	-	-
		1	0.04 (-0.04 - 0.11)	1.09 (0.92 - 1.30)	0.30
		2	0.05 (-0.05 - 0.15)	1.12 (0.89 - 1.41)	0.34
		> 2	0.22 (0.13 - 0.31)	1.67 (1.35 - 2.06)	2.2.E-06
MM	Sex	Female (Ref)	-	-	-
		Male	0.08 (0.04 - 0.12)	1.20 (1.10 - 1.30)	6.3.E-05
	R-ISS	SMM (Ref)	-	-	-
		Low	0.14 (0.05 - 0.23)	1.38 (1.12 - 1.69)	2.2.E-03
		Intermediate	0.37 (0.32 - 0.43)	2.37 (2.10 - 2.68)	4.7.E-44
		High	0.67 (0.61 - 0.74)	4.72 (4.1 - 5.44)	7.9.E-102
	Recent Antimicrobials	0 (Ref)	-	-	-
		1	-0.01 (-0.05 - 0.04)	0.98 (0.88 - 1.09)	0.68
		2	0.06 (0.00 - 0.12)	1.15 (1.00 - 1.31)	0.05
		> 2	0.07 (0.00 - 0.13)	1.16 (1.01 - 1.34)	0.04
DLBCL	Sex	Female (Ref)	-	-	-
		Male	0.08 (0.05 - 0.11)	1.21 (1.12 - 1.3)	2.2.E-06
	R-IPI	Low (Ref)	-	-	-
		Intermediate	0.41 (0.26 - 0.55)	2.55 (1.83 - 3.56)	3.5.E-08
		High	0.70 (0.55 - 0.84)	4.99 (3.57 - 6.98)	4.5.E-21
	Recent Antimicrobials	0	-	-	-
		1	0.02 (-0.02 - 0.06)	1.04 (0.95 - 1.14)	0.42
		2	0.05 (-0.01 - 0.10)	1.11 (0.99 - 1.25)	0.09
		> 2	0.15 (0.09 - 0.20)	1.40 (1.24 - 1.58)	5.8.E-08
FL	Sex	Female (Ref)	-	-	-
		Male	0.18 (0.11 - 0.26)	1.52 (1.28 - 1.81)	2.0.E-06
	FLIPI2	Low (Ref)	-	-	-
		Intermediate	0.17 (-0.01 - 0.35)	1.48 (0.98 - 2.21)	0.06
		High	0.37 (0.19 - 0.56)	2.36 (1.54 - 3.60)	7.6.E-05
	Recent Antimicrobials	0	-	-	-
		1	0.05 (-0.04 - 0.14)	1.11 (0.91 - 1.37)	0.30
		2	0.07 (-0.06 - 0.19)	1.17 (0.88 - 1.55)	0.29
		> 2	0.17 (0.03 - 0.30)	1.47 (1.08 - 2.00)	0.01

**MZL**	**Sex**	Female (Ref)	-	-	-
		Male	0.26 (0.16 - 0.37)	1.84 (1.43 - 2.36)	1.9.E-06
	**MALT-IPI**	Low (Ref)	-	-	-
		Intermediate	0.19 (-0.03 - 0.40)	1.53 (0.94 - 2.52)	0.09
		High	0.27 (0.04 - 0.50)	1.86 (1.10 - 3.15)	0.02
	**Recent Antimicrobials**	0	-	-	-
		1	0.02 (-0.12 - 0.16)	1.05 (0.77 - 1.45)	0.75
		2	0.21 (0.05 - 0.36)	1.61 (1.13 - 2.30)	8.6.E-03
		> 2	0.18 (0.03 - 0.34)	1.53 (1.08 - 2.17)	0.02

**LPL**	**Sex**	Female (Ref)	-	-	-
		Male	0.11 (0.01 - 0.21)	1.29 (1.02 - 1.63)	0.04
	**rIPSSWM**	Very low	0.02 (-0.23 - 0.26)	1.04 (0.59 - 1.83)	0.90
		Low (Ref)	-	-	-
		Intermediate	0.12 (-0.04 - 0.28)	1.32 (0.91 - 1.92)	0.14
		High	0.33 (0.15 - 0.51)	2.13 (1.40 - 3.23)	3.8.E-04
		High high	0.51 (0.30 - 0.71)	3.20 (1.98 - 5.18)	2.1.E-06
	**Recent Antimicrobials**	0 (Ref)	-	-	-
		1	-0.04 (-0.16 - 0.08)	0.91 (0.69 - 1.19)	0.49
		2	-0.02 (-0.18 - 0.15)	0.96 (0.66 - 1.40)	0.84
		> 2	0.10 (-0.05 - 0.25)	1.27 (0.90 - 1.78)	0.18
